# Une hanche douloureuse révélatrice d'une histiocytose osseuse multifocale

**DOI:** 10.11604/pamj.2014.17.105.2652

**Published:** 2014-02-12

**Authors:** Kamal Lahrach, Adil Alaoui, Khalid Ibn el Kadi, Amine Marzouki, Fawzi Boutayeb

**Affiliations:** 1 Service de chirurgie orthopédique et traumatologique (A), Centre hospitalier universitaire Hassan-II, Fès, Maroc

**Keywords:** Histiocytose langerhansienne, hanche, Langerhans cell histiocytosis, hip

## Abstract

L'histiocytose langerhansienne est une maladie rare qui touche principalement l'enfant et l'adulte jeune. Elle peut prendre plusieurs aspects, L'atteinte osseuse peut être uni- ou multifocale. Nous rapportons une observation d'histiocytose langerhansienne osseuse multifocale, révélée chez un patient âgé de 23 ans et ayant touché le col fémoral droit. La scintigraphie osseuse a permis de retrouver plusieurs localisations: l'os temporal, humérale et scapulaire droite. En raison du risque fracturaire, le patient a bénéficié d'une ostéosynthèse par vis-plaque DHS avec curetage biopsie de la lésion. L'examen histologique a révélé une histiocytose langerhansienne. L'évolution fut favorable après chimiothérapie par voie générale.

## Introduction

L'histiocytose langerhansienne (HCL), antérieurement connue sous le nom d'histiocytose X, est une affection diffuse ou localisée du Système Mononuclé Phagocytaire, d′étiologie inconnue, caractérisée par un grand polymorphisme clinique et par une évolution prolongée et incertaine [1]. Il s'agit d'une affection rare, 1 à 2/100 000, touchant surtout l'enfant avec une prédominance masculine [2]. Les localisations osseuses sont les plus fréquentes (60 à 90%) [3]. Le but de ce travail est d′essayer de systématiser la conduite thérapeutique et de définir la place du chirurgien orthopédiste dans le traitement de certaines localisations osseuses de l′histiocytose langerhansienne.

## Patient et observation

**Figure 1:**
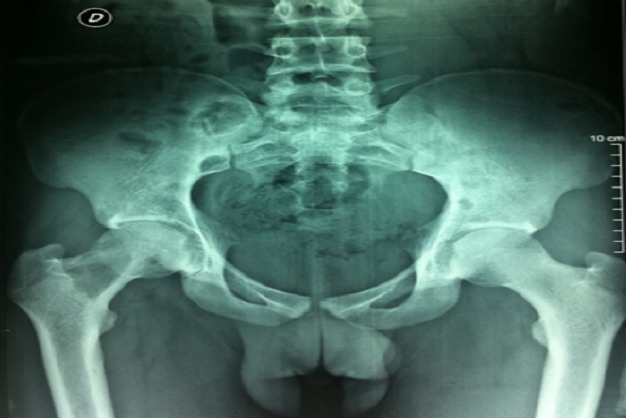
Radiographie du bassin a montré la présence d'une image lytique du col fémoral droit avec respect de la corticale

**Figure 2:**
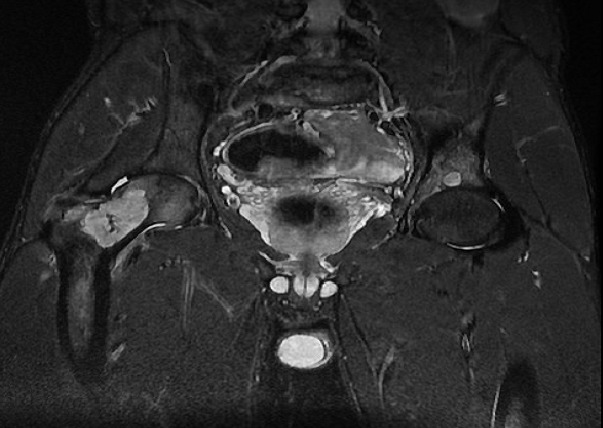
L'imagerie par résonnance magnétique précisant l'étendue de la lacune au niveau du col fémoral

**Figure 3:**
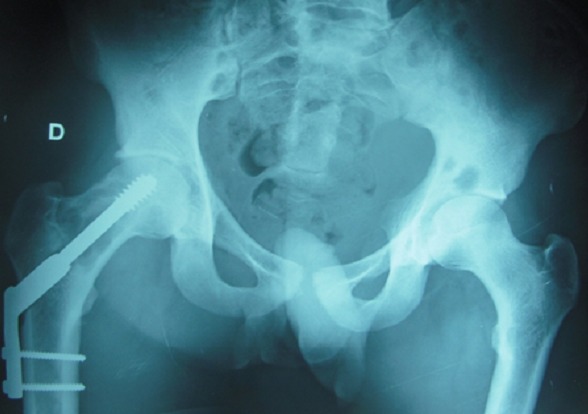
Ostéosynthèse par vis plaque DHS après curetage biopsie de la lacune osseuse

Monsieur A, âgé de 23 ans, d′origine marocaine, sans aucun antécédent pathologique, a été hospitalisé au CHU Hassan II pour douleurs de la hanche droite. Devant ce tableau, il avait reçu un traitement symptomatique dans un centre régional du pays mais sans amélioration. L'examen articulaire a montré une douleur à la mobilisation passive et active de la hanche droite. L'état général du patient était conservé, l'examen clinique ne retrouvait pas de syndrome tumoral, l'examen neurologique ainsi que l'examen cutané et le bilan biologique étaient normaux. La radiographie du bassin a montré la présence d'une image lytique du col fémoral droit avec respect de la corticale (Figure 1). La radiographie du crâne a montré une image d'ostéolyse au niveau de l'os temporal et les radiographies du squelette ont montré une image lytique du tiers supérieur de l'humérus droit. La résonance magnétique a permis de définir avec précision l′étendue de la lacune osseuse (Figure 2). Une scintigraphie osseuse avait révélé une hyperfixation de la hanche droite, de l'extrémité supérieure de l'humérus droit et du crane. L′ostéolyse importante du col fémoral a fait craindre une fracture au moment de la biopsie. L′indication d'une ostéosynthèse par vis plaque DHS a alors été retenue (Figure 3). L'examen anatomopathologique de la biopsie chirurgicale a mis en évidence une prolifération d'histiocytes dérivant des cellules de Langerhans. Le bilan d'extension a compris une tomodensitométrie thoraco-abdominale était normale. Le patient a été traité par corticothérapie à la dose de 1 mg/kg par jour en association avec des cures hebdomadaires de vinblastine à la dose de 4 mg pendant huit semaines. L'évolution fut favorable après chimiothérapie par voie générale. Le recul actuel est de 18 mois.

## Discussion

L′histiocytose X est une maladie caractérisée par une prolifération et une infiltration de cellules histiocytaires dont le comportement pourrait être tantôt celui d′une tumeur osseuse bénigne, tantôt celui d′une “hémopathie maligne”. La cellule d′origine est certainement la cellule de Langerhans et c′est pourquoi actuellement le terme d′histiocytose X a été remplacé par le terme de “Histiocytose à cellules de Langerhans” proposé par Nesbit [4]. Elle atteint plus fréquemment l′adulte jeune, mais elle peut se voir aussi chez le tout petit, exceptionnellement chez l'adulte. Dans notre observation, l'enfant avait 23 ans. L′homme semble plus souvent atteint que la femme [2]. La pathogénie de cette affection reste encore inconnue, même s′il semble s′agir d′un désordre de la régulation immunitaire plutôt que d′un processus néoplasique [5]. Au cours de l'HL, l'atteinte osseuse ostéolytique constitue la circonstance de découverte la plus fréquente. Elle peut se manifester par des douleurs, comme dans notre observation, des fractures et des tuméfactions [6, 7]. Les lésions cutanées sont d'un apport diagnostique important [8]. Les facteurs de mauvais pronostic sont un âge jeune au moment du diagnostic initial et la diffusion rapide de la maladie vers d'autres organes [9]. L'attitude thérapeutique dans les formes osseuses dépend des sites et du nombre de localisations. Les formes uni focales (os, ganglions ou peau) généralement bénignes nécessitent une prise en charge limitée. Les lésions osseuses, uniques ou peu nombreuses ne nécessitent généralement aucun traitement, en dehors de la biopsie ou de la cytoponction nécessaire pour confirmer le diagnostic. Un traitement chirurgical (par curetage ou ostéosynthèse) pour réduire le risque de fracture. Les formes multifocales, font appel à la chimiothérapie seule ou associée a une corticothérapie et/ou a une radiothérapie [10, 11]. La chimiothérapie de première ligne est la vinblastine ou l'étoposide. La radiothérapie est discutable en raison du risque de cancer secondaire dans le champ d′irradiation [12]. A doses modérées (10-15 grays), elle peut néanmoins être utile en cas de lésion très étendue et/ou extensive [13]. Broadbent [14] réserve la radiothérapie aux lésions inaccessibles à l′injection locale de corticoïdes et qui menacent des structures vitales telles que la moëlle épinière ou le nerf optique. La surveillance prolongée doit être la règle dans cette pathologie tumorale non maligne [15].

## Conclusion

L'atteinte osseuse au cours de l'histiocytose langerhansienne est l'une des manifestations les plus fréquentes et qui représente le premier signe de la maladie. Elle est souvent de pronostic favorable. La prise en charge passe par un bilan d'extension osseux et viscéral, qui détermine les choix thérapeutiques.
